# Prognosis and predictive value of *KIT* exon 11 deletion in GISTs

**DOI:** 10.1038/sj.bjc.6605117

**Published:** 2009-06-16

**Authors:** J-B Bachet, I Hostein, A Le Cesne, S Brahimi, A Beauchet, S Tabone-Eglinger, F Subra, B Bui, F Duffaud, P Terrier, J-M Coindre, J-Y Blay, J-F Emile

**Affiliations:** 1 EA4340 ‘Epidémiologie et Oncogénesè des tumeurs digestives’, Faculté de médecine PIFO, UVSQ, 9 boulevard d'Alembert, 78280 Guyancourt, France; 2 Service de Gastroentérologie et Oncologie Digestive, Hôpital Ambroise Paré, APHP, 9 avenue Charles de Gaulle, 92100 Boulogne, France; 3 Service de Génétique Tumorale, Institut Bergonié, 229 Cours de l'Argonne, 33076 Bordeaux, France; 4 Service d'Oncologie, Institut Gustave Roussy, 39 rue Camille Desmoulins, 94805 Villejuif, France; 5 Service d'Anatomo-cyto-pathologie, Hôpital Ambroise Paré, APHP, 9 avenue Charles de Gaulle, 92100 Boulogne, France; 6 Unité de recherche clinique, Hôpital Ambroise Paré, APHP, 9 avenue Charles de Gaulle, 92100 Boulogne, France; 7 INSERM U590, Centre Léon Bérard, 28 rue Laennec, 69373 Lyon Cedex 08, France; 8 LBPA – UMR 8113, école normale supérieure de Cachan 61 avenue du Président Wilson, 94235 Cachan cedex, France; 9 Service de Médecine, Institut Bergonié, 229 Cours de l'Argonne, 33076 Bordeaux, France; 10 Service d'Oncologie médicale, CHU la Timone, APHM, 13385, Marseille cedex 05, France; 11 Service d'Anatomo-cyto-pathologie, Institut Gustave Roussy, 39 rue Camille Desmoulins, 94805 Villejuif, France; 12 Service d'anatomo-cyto-pathologie, Institut Bergonié, 229 Cours de l'Argonne, 33076 Bordeaux, France

**Keywords:** gastrointestinal stromal tumour, GIST, metastasis, prognostic, survival

## Abstract

**Background:**

*KIT* exon 11 mutations are observed in 60% of gastrointestinal stromal tumours (GIST). Exon 11 codes for residues Tyr568 and Tyr570, which play a major role in signal transduction and degradation of KIT. Our aim was to compare the outcome of patients with deletion of both Tyr568–570 (delTyr) and the most frequent deletion delWK557–558 (delWK).

**Methods:**

Pathology and clinical characteristics of 68 patients with delTyr (*n*=26) or delWK (*n*=42) were reviewed and compared.

**Results:**

GISTs with delTyr were more frequently extragastric than those with delWK (69 *vs* 26%, *P*<0.0005). After curative surgery, median relapse-free survival were 10.8 and 11.1 months for patients with delTyr (*n*=14) and delWK (*n*=29), respectively (*P*=0.92). All patients treated with imatinib for a non-resectable or metastatic GIST had an objective response (*n*=15) or a stable disease (*n*=21) as best response, regardless of mutation. Median progression-free survival with imatinib were 21.9 and 18.9 months for patients with GIST with delTyr (*n*=14) and delWK (*n*=22), respectively (*P*=0.43).

**Conclusion:**

In this large retrospective series, the type of *KIT* exon 11 mutation was correlated with the origin of GIST, but not with prognosis or response to imatinib.

Gastrointestinal stromal tumours (GISTs) are the most frequent mesenchymal tumours of the digestive tract and occur typically in the stomach for two-third or in the small intestine for 25% in most series (Emile *et al*, 2004). Gain of function mutations of either *KIT* or platelet-derived growth factor receptor alpha polypeptide (*PDGFRA*) receptor tyrosine kinases play a critical role in GIST pathogenesis, and are found in 85% of GISTs (Rubin *et al*, 2007).

Many types of gain of function mutations of *KIT* and *PDGFRA* have been described in GISTs, but 60% occurred within the exon 11 of *KIT* (Corless *et al*, 2004; Emile *et al*, 2004), which comprises 33 codons (codons 550–582). The two tyrosines Tyr568 and Tyr570, first residues to be phosphorylated during activation, are consensus sites for binding of Src family kinases and could be implicated in activation of different signalling pathways (Roskoski, 2005). More than 90 mutations of exon 11 have been published, and consist in insertions, substitutions and deletions; however, delWK557–558, in the proximal part of exon 11, is the most frequent, accounting for 8–25% of *KIT* exon 11 mutations. Others deletions, in the distal part of the exon, include in particular deletions of Tyr568 and/or Tyr570, and may thus have more specific effects on KIT signalling pathways and degradation. Such deletions account for 3–8% of exon 11 mutations in published series (Ernst *et al*, 1998; Taniguchi *et al*, 1999; Debiec-Rychter *et al*, 2004, 2006; Wardelmann *et al*, 2004; Martin *et al*, 2005; Penzel *et al*, 2005; Andersson *et al*, 2006; Emile *et al*, 2006; DeMatteo *et al*, 2008).

After surgical resection, the type of *KIT* mutations may be a prognostic factor of relapse. *KIT* exon 11 deletions and deletions affecting codons 557–558 of *KIT* exon 11 were described to be independent adverse prognostic factors in patients with GIST (Wardelmann *et al*, 2003; Martin *et al*, 2005; DeMatteo *et al*, 2008). Conversely, in another study, GISTs in which the last part of exon 11 (codons 562–579) was deleted were most frequently associated with malignancy than GISTs with deletion of the first part of exon 11 (codons 550–561; Emile *et al*, 2004, 2006). So, the prognostic value of some types of *KIT* exon 11 mutations for risk of relapse is still debated. The mutational status of *KIT* or *PDGFRA* is predictive of clinical response to imatinib (Glivec, Gleevec, Novartis, Basel, Switzerland) and best results are obtained in patients with GISTs harbouring *KIT* exon 11 mutations (Heinrich *et al*, 2003; Debiec-Rychter *et al*, 2006). Nevertheless, the role of the type of *KIT* exon 11 mutations for the response and survival under imatinib remains to be determined.

Thus, to better understand the prognostic significance of the type of *KIT* exon 11 deletions, we have compared the clinical characteristics and outcome of patients with GIST and deletion of both Tyr568 and Tyr570 with the most frequent deletion of *KIT* exon 11, delWK557–558.

## Materials and methods

### Patient selection

From database of two French pathology departments which detect *KIT* and *PDGFRA* mutations in routine practice (Ambroise Pare Hospital, Boulogne; Bergonie Institute, Bordeaux, France), we searched retrospectively for all consecutive patients with GIST and with either delWK or deletions including both residues Tyr568 and Tyr570 (delTyr). Mutations within exon 9, 11, 13 and 17 of *KIT* and within exon 12 and 18 of *PDGFRA* were detected as previously described (Emile *et al*, 2002, 2004; Heinrich *et al*, 2003; Hostein *et al*, 2006).

### Pathology

All samples were obtained before treatment with imatinib. Paraffin-embedded samples were independently analysed by at least two pathologists. For resected GIST, largest tumour diameter and mitotic count per 50 high-power fields (HPF) were evaluated after surgery in each case, as recommended by international criteria and used to evaluate the risk of GIST malignancy (Fletcher *et al*, 2002; Miettinen and Lasota, 2006). Immunohistochemistry was performed with anti-CD117 (A-4502, polyclonal; DAKO, Copenhagen, Denmark).

### Clinical data and survival analysis

Medical records of all patients were retrospectively reviewed. Response rates to imatinib were evaluated by spiral computerised tomography according to the RECIST criteria. The relapse-free survival (RFS) was defined as the time between the date of curative surgery and the date of relapse. The progression-free survival (PFS) was defined as the time between the first day of imatinib and the date of progression or death. Overall survival (OS) under imatinib was defined as the time between the first day of imatinib and the date of death or last follow-up.

### Statistical analysis

Results are expressed as medians and ranges. The cut-off date for the final analysis was 15 January 2008. We used Student's *t*-test to compare quantitative data in univariate analyses and *χ*^2^-tests were used for qualitative data. We estimated RFS, PFS and OS using the Kaplan–Meier method, and we used log-rank tests to compare the survival curves (Kaplan and Meier, 1958). SAS software v 9.1 (SAS Institute Inc., Cary, NC, USA) was used for all statistical analysis.

## Results

### Mutation, clinical and pathologic characteristics

**Figure 1:**
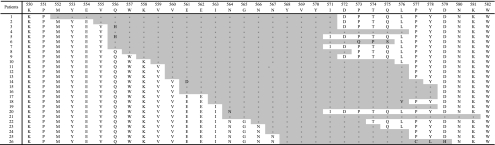
Type and position of deletions including both tyrosines Tyr568 and Tyr570.

A total of 68 patients with GIST, diagnosed between 1985 and 2007, and all CD117 positive, were retrieved. DelWK and delTyr accounted for 18% (34/185) and 10% (19/185) of *KIT* exon 11 mutations in Ambroise Paré's series, respectively. Out of the 26 delTyr mutations, 8 also involved both amino acids 557 and 558, and one involved the amino acid 558. Details of delTyr mutations are summarised in Figure 1.

GISTs with delTyr were more frequently extragastric than those with delWK (69 *vs* 26%, *P*<0.0005), whereas other clinical and tumour characteristics were not different (Table 1). After exclusion of the 8 GISTs with delTyr including the 2 amino acids 557 and 558, GISTs with delTyr (*n*=18) were still more frequently extragastric (*P*=0.0031).

**Figure 2:**
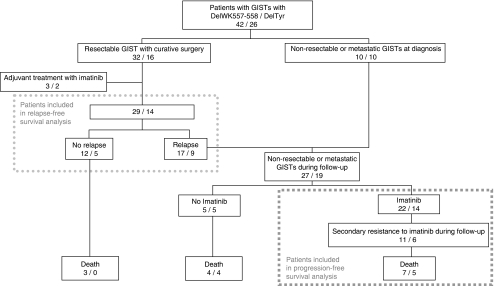
Distribution of the patients according to the outcome.

Distribution of patients according to the outcome and the type of *KIT* exon 11 deletion is described in Figure 2.

### Relapse-free survival

**Figure 3:**
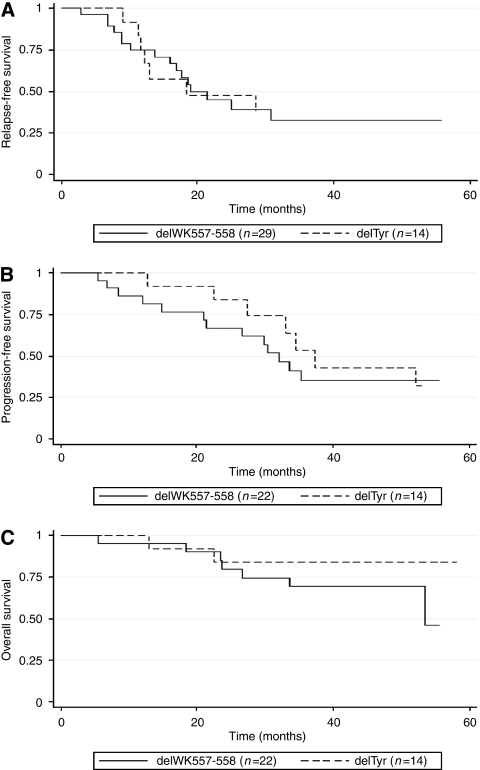
(**A**) Relapse-free survival according to the type of exon 11 deletion. (**B**) Progression-free survival under imatinib according to the type of exon 11 deletion. (**C**) Overall survival under imatinib according to the type of exon 11 deletion.

Mitotic count, tumour size and risk classifications were not different between patients with delWK and those with delTyr (Table 2). At the date of cut-off, median time since curative surgery was 5.1 years (range 0.4–21.9 years). Three patients with delWK and two patients with delTyr had been included in an adjuvant prospective trial with imatinib, and were excluded of RFS analysis. Median RFS were 11.1 months (95% CI: 9.4–66.6) and 10.8 months (95% CI: 7.1–57.7; *P*=0.92; Figure 3A), respectively. Results were not modified after exclusion of the eight GISTs with delTyr including the two amino acids 557 and 558 (*P*=0.45).

### Objective response and survival under imatinib

During follow-up, 22 patients with delWK and 14 patients with delTyr received imatinib (Figure 2). Out of these 36 patients, 26 (72%) had been included and evaluated in a prospective trial. At the date of cut-off, median time since imatinib beginning was 4.7 years (range 0.7–6.8 years).

Objective responses to imatinib were not different between patients with delWK and those with delTyr (Table 3). Median PFS under imatinib were 18.9 months (95% CI: 12.6– ) for patients with delWK and 21.9 months (95% CI: 16.1–37.4) for patients with delTyr (*P*=0.43; Figure 3B). Median OS since imatinib beginning were 31.4 months (95% CI: 19.7–) and 38.6 months (95% CI: 35.4–45.3; *P*=0.31; Figure 3C), respectively. After exclusion of the 8 GISTs with delTyr including the two amino acids 557 and 558, results were not modified for median PFS (*P*=0.60) and OS (*P*=0.39).

## Discussion

*KIT* exon 11 mutations are present in the majority of GISTs. Many different types of mutations have been published, some of which delete residues Tyr568 and Tyr570, which play an important role in KIT signal transduction. Thus, we compared these mutations (delTyr) with the most frequent mutation of *KIT* exon 11 in GISTs (delWK557–558). Analysis of our large series of patients shows that GISTs with delWK are mainly gastric, whereas GISTs with delTyr are mainly intestinal. However, GISTs with these mutations had identical prognosis after curative surgery and response to imatinib treatment.

Previous studies described that the GIST's location was associated with type of mutation. GISTs with *KIT* exon 9 mutation arise predominantly in small intestine and colon, and those with *PDGFRA* mutations most often originate from the stomach (Emile *et al*, 2004; Wardelmann *et al*, 2004; Penzel *et al*, 2005). Our results show that GISTs with delTyr arise in small intestine, colon or rectum in about 70% of cases, whereas those with delWK557–558 occur in stomach in about 75% of cases, and this difference was highly significant. This suggests possible different types of oncogenic events driving *KIT* mutations in the different parts of the digestive tract.

Recently, some studies reported that GISTs with delWK557–558 have an increased risk of relapse after curative surgery (Wardelmann *et al*, 2003; Martin *et al*, 2005; DeMatteo *et al*, 2008). In our study, GISTs with delWK557–558 and GISTs with delTyr did not differ for the risk of relapse after curative surgery and both convey a poor prognosis. According to tumour location, independently of the risk stage, a relapse occurred in 56% (14/25) and 75% (3/4) of gastric GISTs with delWK and delTyr, and in 40% (2/5) and 66.7% (6/9) of intestinal GISTs, respectively. So, GISTs with these mutations seem to have the same worse prognosis and gastric GIST with exon 11 mutations may be of the same poor prognosis as small bowel or large bowel GIST actually.

The outcome of non-resectable and metastatic GISTs with delWK and delTyr under imatinib is similar in terms of response rates, PFS and OS. All patients included in our study had an objective response or a stable disease under imatinib, and median PFS were of about 20 months. These results are concordant with results of published phase III studies (Heinrich *et al*, 2003; Debiec-Rychter *et al*, 2006).

In this large retrospective series, the type of *KIT* exon 11 mutation differed according to primary site, with delWK originating from the stomach, whereas those with delTyr from the intestine. However, GISTs with these mutations had the same prognosis after curative surgery and under imatinib.

## Figures and Tables

**Table 1:** Clinical and pathologic characteristics of patients according to the type of exon 11 deletion

**Type of exon 11 deletion**	**DelWK557–558**	**DelTyr**
Number of patients	42	26
		
Age at diagnosis (years)^a^	58 (19–93)	63 (44–86)
		
*Sex*		
Male	22 (52%)	16 (62.5%)
Female	20 (48%)	10 (37.5%)
		
*Stage of the disease at diagnosis*		
Resectable	32 (76%)	16 (61%)
Non-resectable	2 (5%)	1 (4%)
Metastatic	8 (19%)	9 (35%)
		
*Location of the primary tumour*		
Stomach	31 (74%)	8 (31%)
Small Intestine	8 (19%)	15 (58%)
Colon/rectum	3 (7%)	3 (11%)
		
*Histologic phenotype*		
Spindle type	31 (74%)	21 (80%)
Epithelioid type	3 (7%)	1 (4%)
Mixed type	8 (19%)	4 (15%)
		
*Percent of necrosis*		
0%	15 (36%)	9 (35%)
<50%	23 (55%)	9 (35%)
>50%	2 (5%)	6 (23%)
Non-evaluable	2 (5%)	2 (8%)
Positive CD117 staining	42 (100%)	26 (100%)

a Median and range.

**Table 2:** Prognostic factors after curative surgery and outcome of patients according to the type of exon 11 deletion

**Type of exon 11 deletion**	**DelWK557–558**	**DelTyr**
Number of resected GISTs at diagnosis	32	16
		
*Largest tumour diameter (cm)*		
<2	1 (3%)	0
2–5	7 (22%)	6 (37.5%)
5–10	13 (41%)	2 (12.5%)
>10	11 (34%)	8 (50%)
		
*Mitotic count per 50 HPF*		
<5	8 (25%)	8 (50%)
6–10	4 (12.5%)	2 (12.5%)
>10	20 (62.5%)	6 (37.5%)
		
*Risk stage* (Fletcher *et al*, 2002)		
Very low	0	0
Low	2 (6%)	4 (25%)
Intermediate	4 (13%)	2 (12.5%)
High	26 (81%)	10 (62.5%)
		
*Risk stage* (Miettinen and Lasota, 2006)		
Very low	2 (6%)	1 (6%)
Low	3 (9%)	3 (19%)
Intermediate	6 (19%)	1 (6%)
High	20 (63%)	11 (69%)
Non-evaluable	1 (3%)	0
		
*Relapse after curative surgery for patients with a resectable GIST at diagnosis (number of patients)*		
No	15 (47%)	7 (44%)
Yes	17 (53%)	9 (56%)
		
*Relapse location after curative surgery* a		
Peritoneum	13 (76%)	8 (89%)
Liver	5 (29%)	5 (56%)
Lung	1 (6%)	0

a Some patients had two sites of relapse.

**Table 3:** Outcome under imatinib according to the type of exon 11 deletion

**Type of exon 11 deletion**	**DelWK557–558**	**DelTyr**
Treatment by imatinib for patients with a non-resectable or metastatic GIST during follow-up	(*n*=27)	(*n*=19)
No	5 (19%)	5 (26%)
Yes	22 (81%)	14 (74%)
		
*Best response under imatinib (RECIST criteria)*		
Complete response	1 (5%)	1 (7%)
Partial response	9 (41%)	4 (29%)
Stable disease	12 (55%)	9 (64%)
Progressive disease	0	0
		
Number of patients who had secondary resistance to imatinib during follow-up	11 (50%)	6 (43%)
Number of patients dead at the end of follow-up	14 (33%)	10 (38%)
		
*Cause of death*		
GIST	8 (57%)	8 (80%)
Others	6 (43%)	2 (20%)
